# Tumor‐expressed microRNAs associated with venous thromboembolism in colorectal cancer

**DOI:** 10.1002/rth2.12749

**Published:** 2022-07-01

**Authors:** Rayna J. S. Anijs, El Houari Laghmani, Betül Ünlü, Szymon M. Kiełbasa, Hailiang Mei, Suzanne C. Cannegieter, Frederikus A. Klok, Peter J. K. Kuppen, Henri H. Versteeg, Jeroen T. Buijs

**Affiliations:** ^1^ Einthoven Laboratory for Vascular and Regenerative Medicine, Division of Thrombosis and Hemostasis, Department of Medicine Leiden University Medical Center Leiden The Netherlands; ^2^ Department of Clinical Epidemiology Leiden University Medical Center Leiden The Netherlands; ^3^ Department of Biomedical Data Sciences Leiden University Medical Center Leiden The Netherlands; ^4^ Department of Surgery Leiden University Medical Center Leiden The Netherlands

**Keywords:** biomarkers, colorectal neoplasms, high‐throughput nucleotide sequencing, microRNAs, venous thromboembolism

## Abstract

**Background:**

Colorectal cancer patients have an increased risk of developing venous thromboembolism (VTE), resulting in increased morbidity and mortality. Because the exact mechanism is yet unknown, risk prediction is still challenging; therefore, new biomarkers are needed. MicroRNAs (miRNAs) are small, relatively stable RNAs, that regulate a variety of cellular processes, and are easily measured in body fluids.

**Objective:**

The aim of this study was to identify novel tumor‐expressed miRNAs associated with VTE.

**Methods:**

In a cohort of 418 colorectal cancer patients diagnosed between 2001 and 2015 at the Leiden University Medical Center, 23 patients (5.5%) developed VTE 1 year before or after cancer diagnosis. Based on availability of frozen tumor material, tumor cells of 17 patients with VTE and 18 patients without VTE were isolated using laser capture microdissection and subsequently analyzed on the Illumina sequencing platform NovaSeq600 using 150‐bp paired‐end sequencing. Cases and controls were matched on age, sex, tumor stage, and grade. Differential miRNA expression was analyzed using edgeR.

**Results:**

A total of 547 miRNAs were detected. Applying a 1.5‐fold difference and false discovery rate of <0.1, 19 tumor‐miRNAs were differentially regulated in VTE cases versus controls, with hsa‐miR‐3652, hsa‐miR‐92b‐5p, and hsa‐miR‐10,394‐5p as most significantly downregulated. Seven of the 19 identified miRNAs were predicted to regulate the gonadotropin‐releasing hormone receptor pathway.

**Conclusion:**

We identified 19 differentially regulated tumor‐expressed miRNAs in colorectal cancer‐associated VTE, which may provide insights into the biological mechanism and in the future might have potential to serve as novel, predictive biomarkers.

**Essentials**

- microRNAs are promising biomarkers for venous thromboembolism (VTE) in patients with cancer.
- microRNA profiling was performed on colorectal cancer samples from patients with and without VTE.
- 19 tumor‐expressed microRNAs were differentially regulated in patients with VTE.
- 7 of 19 identified microRNAs predicted to regulate Gonadotropin‐releasing hormone receptor pathway.

## 1 INTRODUCTION

Cancer patients have a 9‐fold increased risk of developing venous thromboembolism (VTE) compared with the general population in the first year after diagnosis.^1^, ^2^ The risk is determined by several cancer‐related factors, including chemotherapy, surgery, tumor type, and stage.^2^ Colorectal cancer patients have an intermediate risk of VTE with a 6‐month incidence rate of 5 per 100 person‐years after diagnosis.^3^ Because it is the third most diagnosed cancer type worldwide, with 1.8 million new cases annually, the absolute number of VTE occurrences in these patients is high.^3^

Cancer patients at high risk of VTE may benefit from thromboprophylaxis.^4^, ^5^ Currently, the Khorana risk score, including clinical and laboratory variables, is the only model endorsed for clinical use.^6^, ^7^ However, its performance is suboptimal; therefore, an improved risk stratification is urgently needed.^8^ A better understanding of factors involved in cancer‐associated thrombosis (CAT), may result in identification of novel biomarkers. Consideration of these factors alone or when incorporated in existing risk models could improve risk prediction leading to more targeted treatment of CAT.

MicroRNAs (miRNAs) are a promising class of biomarkers for diagnosis and prediction in both cancer and cardiovascular disease. miRNAs are small, endogenous, single‐stranded, noncoding RNAs, sized ~22 nucleotides that regulate gene expression by stimulating messenger RNA degradation or by inhibiting translation. Each miRNA can regulate numerous messenger RNAs by complementary base‐pairing regulating gene expression at post‐transcriptional level.^9^ miRNAs are expressed intracellularly but are also secreted and detectable in blood and other body fluids. Importantly, miRNAs are relatively stable because of their resistance to nuclease digestion, leading to high reproducibility when measured.^9^, ^10^ Therefore, miRNAs appear to be suitable biomarkers, particularly when assayed in relatively accessible body fluids.^9^ In contrast, obtaining tumor material from miRNAs is more complex, but the tumor‐expressed miRNA profile may better elucidate which tumor‐intrinsic pathways contribute to CAT. Although it was recently shown that certain miRNA plasma levels are associate with CAT development, no studies to date have explored associations between tumor‐expressed miRNAs and CAT.^11^, ^12^, ^13^, ^14^

In this nested case control study, we have compared tumor expressed miRNA profiles from colorectal cancer patients with VTE to those without VTE, which may lead to new biological insights and potentially novel biomarkers for CAT.

## 2 METHODS

**FIGURE 1:**
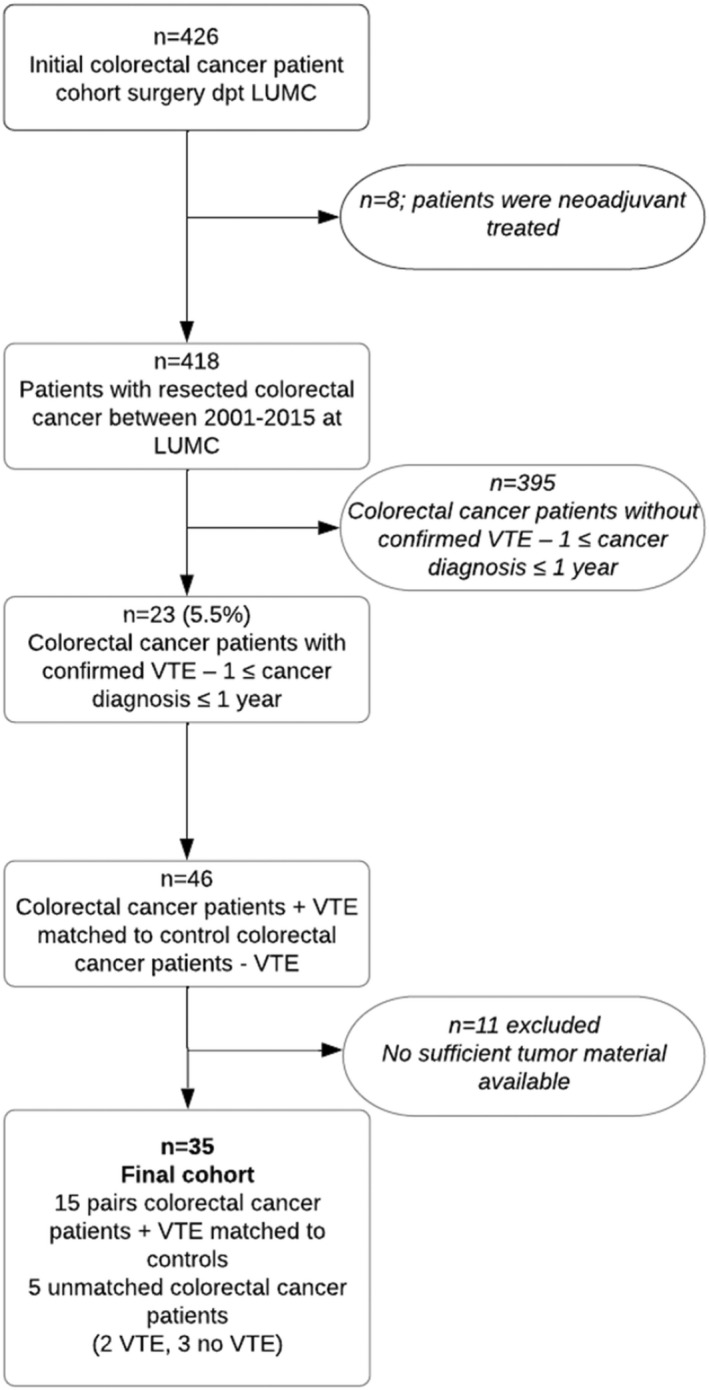
Flow diagram of study design and colorectal cancer patients included. Abbreviations: dpt, department; LUMC, Leiden University Medical Center; VTE, venous thromboembolism

Between January 2001 and December 2015, 418 colorectal cancer patients were identified who underwent curative or palliative surgery at the Leiden University Medical Center (LUMC; Figure 1). VTE events were retrospectively assessed from hospital records and occurred in 23 patients (5.5%) 1 year before or after cancer diagnosis.^15^, ^16^ All 23 cases were individually matched based on sex, tumor stage, grade, and age at cancer diagnosis, to 23 cancer control patients without VTE. From the 46 matched patients, 11 did not have (sufficient) fresh frozen tumor material to be processed for miRNA sequencing. The resulting 35 included patients correspond to 15 fully matched patient couples and five unmatched patients (2× VTE, 3× no VTE; Figure 1). The need for informed consent was waived by the institutional review board of the LUMC because of the retrospective nature of the study and that a large proportion of patients were deceased.

All tumor samples were fresh frozen after resection at the Department of Surgery at the LUMC and stored at −80°C under comparable conditions. Laser capture microdissection (LCM) was used to isolate tumor cells from the whole tumor, based on morphological differences between tumor and stromal cells. Briefly, 7‐μm sections were mounted on Membrane Slide NF 1.0 PEN glasses, rehydrated, hematoxylin‐treated for nuclei staining, washed in 1% ammonia, and finally dehydrated. Sections were processed on the PALM MicroBeam (Zeiss) with an ultraviolet laser and tumor cells were collected into adhesive clear cap tubes. To prevent RNA degradation, tumor slices were subjected to LCM within 60 min after thawing.^17^ LCM tissue from each patient was pooled and small RNAs were isolated using the miRVana kit (ThermoFischer Scientific) according to the manufacturer’s instructions. A fragment analyzer was used to assess the RNA quality before RNA sequencing was started. All 35 samples passed this quality control. The NEBNext Ultra Directional RNA library Prep Kit (Ipswich) for Illumina was used to process the isolated miRNA, after which clustering and miRNA sequencing with the Illumina NovaSeq600 was performed according to the manufacturer's protocol at the sequencing facility GenomeScan B.V. During transfer to GenomeScan, all samples were transferred on dry ice.

The paired‐end raw reads were processed using the BioWDL small‐RNA pipeline version 1.2.0 developed at the LUMC (https://github.com/biowdl/small‐rna). Adapter clipping was performed using Cutadapt (version 2.4) with the forward adapter sequence of “AGATCGGAAGAG” and reverse adapter sequence of “GATCGTCGGACT.” Reads were further aligned to the human reference genome GRCh38 using the Bowtie aligner (version 1.2.2). The following specific settings were used in Bowtie to tackle the error profile and short read length of miRNA sequences: “‐‐seedmms 3 ‐k 3 ‐‐best ‐‐strata.” miRNA read quantification was performed using HTseq‐count (version 0.9.1) based on miRBase miRNA annotation (version 22; http://www.mirbase.org/). After Trimmed Mean of M‐values (TMM) normalization, the differential gene expression analysis was performed with EdgeR 3.14.0.

To exclude any effect of cancer‐related treatment on VTE, a subgroup analysis was performed on VTE patients in the year before cancer diagnosis (four pairs).

Data were analyzed using R‐studio version 4.0.2. The Benjamini‐Hochberg method was used to adjust *p* values for a false discovery rate (FDR). As the aim here was an exploratory screening, a predetermined statistical significance cutoff of <0.1 was used and a fold change (FC) of 1.5 was considered to represent a meaningful biological effect.

## 3 RESULTS AND DISCUSSION

The aim of this nested case–control study was to identify novel tumor‐expressed miRNAs associated with VTE in colorectal cancer. At baseline, cases were on average 68.4 years and 47.1% were male, whereas controls were 69.2 years and 44.4% were male (Table 1). Of all observed VTE events in the cases, pulmonary embolism was most commonly diagnosed (47%), followed by deep vein thrombosis (23.5%) and venous thrombosis in the inferior vena cava (5.9%). The remaining events were CAT at other sites (portal vein, mesenteric vein, jugular vein, or ovarian vein). A total of 29.4% of the cases and 33.3% of the controls had a sigmoid colon tumor. Thirteen patients developed VTE after cancer and four developed VTE before cancer (see also Table S1 for more detailed information on the individually matched patient couples).

**TABLE 1:** Baseline characteristics of the study population

	Overall	Cases (VTE)	Controls (no VTE)
Sex n (%)			
Male	16 (45.7)	8 (47.1)	8 (44.4)
Female	19 (54.3)	9 (52.9)	10 (55.6)
Age (mean SD), y	68.8 (9.50)	68.4 (11.0)	69.2 (8.17)
Anatomic tumor site, *n* (%)			
Cecum	9 (25.7)	4 (23.5)	5 (27.8)
Ascending colon	5 (14.3)	3 (17.6)	2 (11.1)
Sigmoid colon	11 (31.4)	5 (29.4)	6 (33.3)
Transverse colon	5 (14.3)	2 (11.8)	3 (16.7)
Hepatic flexure	2 (5.7)	2 (11.8)	0 (0)
Lienalis flexure	1 (2.9)	0 (0)	1 (5.6)
Rectosigmoid	1 (2.9)	1 (5.9)	0 (0)
Pathological TNM stage, *n* (%)			
1	10 (28.6)	4 (23.5)	6 (33.3)
2	6 (17.1)	3 (17.6)	3 (16.7)
3	8 (22.9)	5 (29.4)	3 (16.7)
4	11 (31.4)	5 (29.4)	6 (33.3)
Tumor grade, *n* (%)			
1	10 (28.6)	4 (23.5)	6 (33.3)
2	6 (17.1)	3 (17.6)	3 (16.7)
3	10 (28.6)	6 (35.3)	4 (22.2)
4	9 (25.7)	4 (23.5)	5 (27.8)
Location of VTE, *n* (%)			
DVT	–	4 (23.5)	–
PE	–	8 (47.0)	–
Inferior vena cava	–	1 (5.9)	–
Jugular vein	–	1 (5.9)	–
Mesenteric vein	–	1 (5.9)	–
Ovarian vein	–	1 (5.9)	–
Portal vein	–	1 (5.9)	–

*Note*: Grade = histological grade with G1: well‐differentiated, G2: moderately differentiated, G3: poorly differentiated, G4 undifferentiated.^26^

Abbreviations: DVT, deep vein thrombosis; M, presence of distant metastasis; N, spread to regional lymph nodes; PE, pulmonary embolism, pathological; TNM staging, tumor classification used according American Joint Committee on Cancer (AJCC) with T: size or direct extent of the primary tumor; VTE, venous thromboembolism.

**FIGURE 2:**
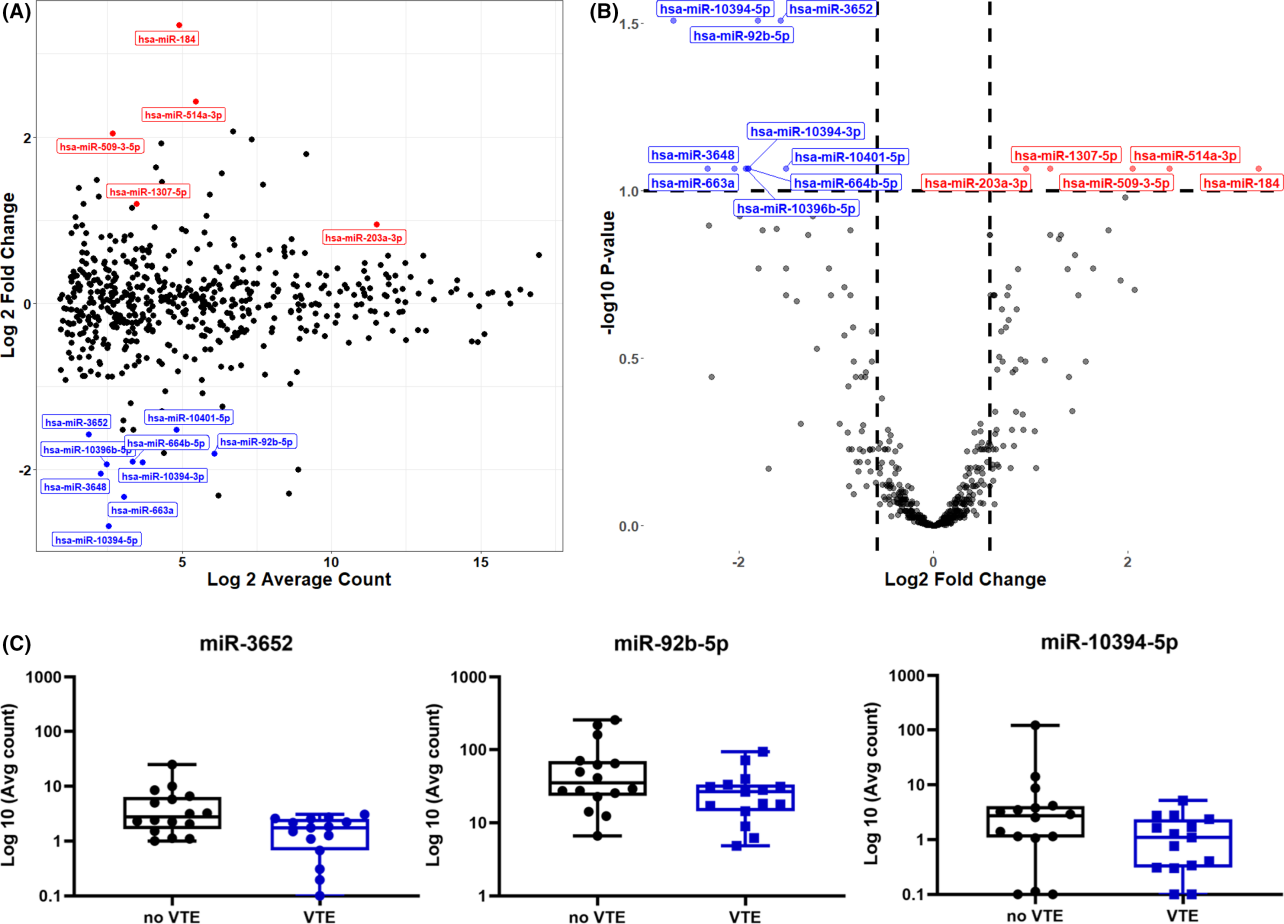
Differential expression of miRNAs in tumor tissue from colorectal cancer patients with VTE compared with those without VTE. (A) The MA Bland‐Altman plot shows for each miRNA the values of the log2 fold change (M‐value) against the average abundance or count (A‐value); red and blue indicate the statistically significant miRNAs that were up‐ and downregulated, respectively. (B) The volcano plot illustrates the distribution of individual log10 *p* values and log2‐fold changes of analyzed miRNAs in the main analysis; red indicates miRNAs significantly upregulated and blue indicates miRNAs significantly downregulated. (C): Individual boxplots of the top 3 differentially downregulated miRNAs in the main analysis. Abbreviations: miRNA, micro RNA; VTE, venous thromboembolism

In the primary analysis, tumor tissue from 17 cases was compared with tissue of 18 controls. From a total of 547 analyzed miRNAs, after correcting for multiple testing, 14 were differentially expressed between cases and controls (Figure 2A,B). Figure 2A shows a MA Bland–Altman plot, visualizing gene expression changes in terms of log2 FC on the Y‐axis (M‐values) versus log2 of mean normalized expression counts on the X‐axis (A‐values). The volcano plot in Figure 2B shows log2 FC versus statistical significance (*p* value). Table 2 summarizes all differentially regulated miRNAs ranked from lowest to highest *p* value and FC differences on linear scale. Hsa‐miR‐3652, hsa‐miR‐92b‐5p, hsa‐miR‐10,394‐5p, hsa‐miR‐3648, hsa‐miR‐10,394‐3p, hsa‐miR‐10,401‐5p, hsa‐miR‐10396b‐5p, hsa‐miR‐663a, and hsa‐miR‐664b‐5p were downregulated, whereas hsa‐miR‐184, hsa‐miR‐509‐3‐5p, hsa‐miR‐1307‐5p, hsa‐miR‐514a‐3p, and hsa‐miR‐203a‐3p were upregulated in cases versus controls. Of all differentially regulated miRNAs, miR‐203a‐3p showed the highest absolute expression (Figure 2A). miR‐3652, miR‐92b‐5p, and miR‐10,394‐5p were most significantly regulated, with an adjusted *p* value of 0.03 and FCs of 0.34, 0.29, and 0.16, respectively (Figure 2C). In the subgroup analysis, with VTE occurring before cancer, seven miRNAs were differentially regulated; hsa‐miR‐10,394‐3p, hsa‐miR‐miR‐483‐5p, hsa‐miR‐10,394‐5p, hsa‐miR‐182‐5p, and hsa‐miR‐3654 were downregulated, and hsa‐miR‐223‐3p and hsa‐miR‐363‐3p were upregulated (Table 2). Notably, miR‐10,394‐3p and miR‐10,394‐5p were also downregulated in the main analysis. This suggests a mechanistic role for these miRNAs in CAT because the patients were at this point unaffected by any cancer treatments.

**TABLE 2:** Differentially tumor‐expressed miRNAs in colorectal cancer patients with VTE compared with those without VTE, with their predicted pathways

	Main analysis					
miRNA	Regulation	Average fold change	Average log2 Fold change	*p* value	Adjusted *p* value	Predicted PANTHER pathways
hsa‐miR‐3652	down	0.34	−1.57	1.36e‐5	0.03	Cadherin signaling pathway, apoptosis signaling pathway, Wnt signaling pathway
hsa‐miR‐92b‐5p	Down	0.29	−1.81	1.64e‐5	0.03	De novo purine biosynthesis, FGF signaling pathway, gonadotropin‐releasing hormone receptor pathway
hsa‐miR‐10,394‐5p	Down	0.16	−2.68	1.71e‐5	0.03	MYO signaling pathway, GBB signaling pathway, ALP23B signaling pathway
hsa‐miR‐184	Up	10.1	3.34	9.56e‐5	0.09	Flavin biosynthesis, TCA cycle, androgen/estrogen/progesterone biosynthesis
hsa‐miR‐3648	Down	0.24	−2.05	9.81e‐5	0.09	2‐arachidonoylglycerol biosynthesis, PI3 kinase pathway, Alzheimer disease‐amyloid secretase pathway
hsa‐miR‐10,394‐3p	Down	0.27	−1.91	1.25e‐4	0.09	Alzheimer disease‐amyloid secretase pathway, gonadotropin‐releasing hormone receptor pathway, alpha adrenergic receptor signaling pathway
hsa‐miR‐10,401‐5p	Down	0.35	−1.52	1.51e‐4	0.09	Threonine biosynthesis, thiamin metabolism, acetate utilization
hsa‐miR‐509‐3‐5p	Up	4.13	2.05	1.64e‐4	0.09	Pyridoxal phosphate salvage pathway, vitamin B6 metabolism, formyltetrahydroformate biosynthesis
hsa‐miR‐1307‐5p	Up	2.30	1.20	1.85e‐4	0.09	Wnt signaling pathway, inflammation mediated by chemokine and cytokine signaling pathway, gonadotropin‐releasing hormone receptor pathway
hsa‐miR‐10,396b‐5p	Down	0.26	−1.93	1.87e‐4	0.09	Muscarinic acetylcholine receptor 1 and 3 signaling pathway, gonadotropin‐releasing hormone receptor pathway, metabotropic glutamate receptor group III pathway
hsa‐miR‐663a	Down	0.20	−2.32	1.98e‐4	0.09	Metabotropic glutamate receptor group III pathway, gonadotropin‐releasing hormone receptor pathway, TGF‐beta signaling pathway
hsa‐miR‐664b‐5p	Down	0.27	−1.90	2.00e‐4	0.09	Regulation of primary metabolic process, regulation of nitrogen compound metabolic process, regulation of cellular metabolic process
hsa‐miR‐514a‐3p	Up	5.38	2.42	2.04e‐4	0.09	p53 pathway feedback loops 2, FGF signaling pathway, EGF receptor signaling pathway
hsa‐miR‐203a‐3p	Up	1.94	0.95	2.19e‐4	0.09	Interferon‐gamma signaling pathway, Ras pathway, insulin/IGF pathway‐protein kinase B signaling cascade
Subgroup VTE before cancer diagnosis						
hsa‐miR‐10,394‐3p	Down	0.03	−5.00	2.83e‐5	0.09	Alzheimer disease‐amyloid secretase pathway, gonadotropin‐releasing hormone receptor pathway, alpha adrenergic receptor signaling pathway
hsa‐miR‐483‐5p	Down	0.01	−6.47	3.30e‐5	0.09	Cysteine biosynthesis, angiotensin II‐stimulated signaling through G proteins and beta‐arrestin, VEGF signaling pathway
hsa‐miR‐10,394‐5p	Down	0.01	−6.76	5.83e‐5	0.097	MYO signaling pathway, GBB signaling pathway, ALP23B signaling pathway
hsa‐miR‐182‐5p	Down	0.29	−1.84	8.82e‐5	0.097	PI3 kinase pathway, EGF receptor signaling pathway, gonadotropin‐releasing hormone receptor pathway
hsa‐miR‐3654	Down	0.05	−4.29	1.13e‐4	0.097	Cytoskeletal regulation by Rho GTPase, Parkinson disease, axon guidance mediated by netrin
hsa‐miR‐223‐3p	Up	16.1	4.01	1.24e‐4	0.097	Insulin/IGF pathway‐protein kinase B signaling cascade, CCKR signaling, gonadotropin‐releasing hormone receptor pathway
hsa‐miR‐363‐3p	Up	7.21	2.85	1.24e‐4	0.097	Integrin signaling pathway, oxidative stress response, N‐acetylglucosamine metabolism

*Note*: Underlined miRNAs were identified in both analyses.

Abbreviation: VTE, venous thromboembolism.

One miRNA in our panel, miR‐483‐5p, was previously identified in a noncancer thrombosis setting. miR‐483‐3p, its minor miRNA arm, was upregulated in endothelial cells from deep vein thrombosis patients compared with control cells and its overexpression diminished thrombus resolution in vivo.^18^ However, in the current study, miR‐483‐5p was downregulated in tumor cells of VTE patients, pointing toward a different role for miR‐483‐5p in endothelial versus tumor cells in thrombosis.

To date, two studies addressed a role for miRNAs in CAT. In pancreatic ductal adenocarcinoma and distal extrahepatic cholangiocarcinoma patients, 11 plasma miRNAs associated with VTE, but none of these were identified in our study.^11^ In another study, six upregulated plasma miRNAs from glioma patients predicted the risk of early postsurgical pulmonary embolism.^12^ Interestingly, miR‐363‐3p, also identified in the current study, was one of the six.^12^ Furthermore, in a conference abstract presented by the same research group, miR‐363‐3p was also increased in plasma from lung CAT patients.^13^ It is tempting to speculate that the increased levels of miR‐363‐3p observed in plasma from CAT patients are a result of increased expression and secretion of miR‐363‐3p by tumor cells. However, it remains to be investigated whether miR‐363‐3p is also upregulated in plasma from colorectal cancer patients with VTE, and whether miR‐363‐3p is also differentially regulated in pancreatic, cholangiocarcinoma, glioma, and lung cancer cells of VTE patients. In addition, increased miR‐363‐3p plasma levels might be caused in part by higher expression and secretion by noncancerous cells as well, reflecting a more general hypercoagulable state. In cancer, miR‐363‐3p was described to regulate integrin and ERK1/2‐signaling, but whether these pathways are used to exert thrombogenic effects is unknown.^19^, ^20^

Besides miR‐363‐3p, there was no overlap of identified miRNAs between the current study and those described previously. This could be explained by two major differences between our study and the two published reports, which were tumor type (colorectal cancer vs. glioma, pancreatic, and cholangiocarcinoma) and type of sample (tumor tissue vs. plasma). Regardless of differences in study design, the finding that miR‐363‐3p was upregulated in plasma from glioma and lung cancer patients and in colorectal tumor tissue in patients with VTE, is of interest and suggestive of a causal role for miR‐363‐3p in CAT.

To explore pathways through which the identified miRNAs may exert prothrombotic effects, the top three predicted pathways of all miRNAs were identified using miRPathDB v2.0. and PANTHER pathways algorithm (Table 2). For many miRNAs, pathways known to be involved in cancer were found, such as p53, Wnt, apoptosis, and Ras signaling.^21^ Seven of the 19 identified miRNAs were predicted to regulate the gonadotropin‐releasing hormone receptor pathway. Interestingly, activation of gonadotropin‐releasing hormone receptor caused G‐protein αi‐mediated activation of phospho‐tyrosine phosphatase, resulting in dephosphorylated EGF receptor (EGFR) and inhibited EGFR signal transduction through the phosphorylated ERK1/2 or the phosphorylated PI3K/AKT pathway.^22^, ^23^, ^24^ EGFR‐signaling has consistently been found to upregulate tissue factor (TF) in glioma, hepatocellular carcinoma and colorectal cancer cells, providing a potential link to the cause of VTE.^22^, ^23^, ^24^, ^25^ Alternatively, TF can activate the pERK and pAKT pathway and subsequently activate the EGFR pathway, thus creating a positive feedback loop.^25^

Because of our study’s exploratory nature, an FDR cutoff of 0.1 was chosen; therefore, it could be expected that two miRNAs (~10% of the 19 identified miRNAs) may be false‐positive and 17 (~90% of the 19 identified miRNAs) true‐positive. If the more stringent FDR cutoff level of 0.05 was applied, hsa‐miR‐3652, hsa‐miR‐92b‐5p, and hsa‐miR‐10,394‐5p would still be considered significant. Nonetheless, functional validation of all of these miRNAs is warranted in in vitro*,* ex vivo, and in vivo CAT models.

Last, data on race or ethnicity was not available in our cohort; the results from this cohort need to be cautiously interpreted when translated to a cohort in a country with a different distribution of race/ethnicity.

In conclusion, a tumor‐expressed miRNA profile associated with CAT was established, which may contribute to hypercoagulability in colorectal cancer. As follow‐up, external validation of this profile is first needed in a larger colorectal cancer cohort and subsequently experimental validation. Moreover, it remains of utmost interest to investigate whether these tumor‐expressed miRNAs are also differentially regulated in plasma of colorectal cancer patients so that they could be used as potential CAT plasma biomarkers.

## AUTHOR CONTRIBUTIONS

Study design: P.J.K.K., H.H.V., and J.T.B. Data collection: R.J.S.A., E.H.L., B.U., and F.A.K. Data analysis and statistical evaluation: R.J.S.A., S.M.K., H.M., S.C.C., and J.T.B. Data interpretation: R.J.S.A, F.A.K., S.C.C., P.J.K.K, H.H.V., and J.T.B. Writing/approving manuscript: all authors.

## RELATIONSHIP DISCLOSURE

The authors have no conflicts of interest.

## Supporting information


Table S1
