# Supplementary material for: Tumor‐expressed microRNAs associated with venous thromboembolism in colorectal cancer
Source: Res Pract Thromb Haemost. 2022 Jul 1;6(5):e12749. doi: 10.1002/rth2.12749 (PMC9248312; doi:10.1002/rth2.12749)
Supplement: Supplementary file 1 — Table S1 [file RTH2-6-e12749-s001.docx]

**Table S1:** Baseline characteristics of the 15 colorectal cancer patients with VTE and their patient controls individually matched on sex, age and tumor stage, with 5 additional unmatched patients.

| Patients (couple) | Sex | Age (years) | Tumor site | TNM stage | Tumor grade | VTE | Location VTE | Days cancer diagnosis --> Surgery | Days cancer diagnosis --> VTE diagnosis | Days surgery --> VTE diagnosis |
| --- | --- | --- | --- | --- | --- | --- | --- | --- | --- | --- |
| 1A | Male | 61 | Cecum | T3N0M0 | G2 | Yes | PE | 0 | 0 | 0 |
| 1B |  | 62 | Descending colon | T3N0M0 | G2 | No |  |  |  |  |
| 2A | Male | 77 | Transverse colon | T3N0M0 | G2 | Yes | PE | 0 | 6 | 6 |
| 2B |  | 77 | Sigmoid colon | T3N0M0 | G2 | No |  |  |  |  |
| 3A | Female | 64 | Sigmoid colon | T3N1M0 | G3 | Yes | DVT | 25 | 152 | 127 |
| 3B |  | 64 | Cecum | T3N1M0 | G3 | No |  |  |  |  |
| 4A | Male | 58 | Transverse colon | T3N0M1a | G4 | Yes | Inferior vena cava | 32 | 68 | 36 |
| 4B |  | 59 | Not specified | T3N0M1a | G4 | No |  |  |  |  |
| 5A | Male | 83 | Sigmoid colon | T3N0M1a | G4 | Yes | Jugular vein | 31 | 49 | 18 |
| 5B |  | 82 | Transverse colon | T3N0M1a | G4 | No |  |  |  |  |
| 6A | Male | 74 | Hepatic flexure | T3N1aM0 | G3 | Yes | PE | 22 | 130 | 108 |
| 6B |  | 72 | Sigmoid colon | T3N1bM0 | G3 | No |  |  |  |  |
| 7A | Male | 74 | Ascending colon | T3N2a | - | Yes | PE | 18 | 364 | 346 |
| 7B |  | 74 | Cecum | T3N2M0 | G3 | No |  |  |  |  |
| 8A | Male | 69 | Cecum | T3N1BM0 | G3 | Yes | DVT | 44 | 124 | 80 |
| 8B |  | 68 | Lienalis flexure | T3N1M0 | G3 | No |  |  |  |  |
| 9A | Male | 67 | Cecum | T2N0M0 | G1 | Yes | PE | 13 | -173 | -186 |
| 9B |  | 67 | Transverse colon | T2N0M0 | G1 | No |  |  |  |  |
| 10A | Female | 75 | Ascending colon | T3N0M0 | G2 | Yes | DVT | 38 | -6 | -44 |
| 10B |  | 75 | Sigmoid colon | T3N0M0 | G2 | No |  |  |  |  |
| 11A | Female | 76 | Sigmoid colon | T2N0M0 | G1 | Yes | PE | 21 | 26 | 5 |
| 11B |  | 78 | Ascending colon | T2N0M0 | G1 | No |  |  |  |  |
| 12A | Female | 53 | Sigmoid colon | T4aN0M1B | G4 | Yes | Ovarian vein | 0 | 92 | 92 |
| 12B |  | 61 | Ascending colon | T3N0M1 | G4 | No |  |  |  |  |
| 13A | Female | 61 | Transverse colon | T3N2M1 | G4 | Yes | DVT | 12 | 130 | 118 |
| 13B |  | 62 | Transverse colon | T3N2aM1a | G4 | No |  |  |  |  |
| 14A | Female | 68 | Rectosigmoid | T2N0M0 | G1 | Yes | Portal vein | 46 | 75 | 29 |
| 14B |  | 71 | Cecum | T2N0M0 | G1 | No |  |  |  |  |
| 15A | Female | 84 | Hepatic flexure | T2N0M0 | G1 | Yes | PE | 39 | 3 | -36 |
| 15B |  | 83 | Sigmoid colon | T2N0M0 | G1 | No |  |  |  |  |
| 16 | Female | 42 | Ascending colon | T3N2M0 | G3 | Yes | PE | 116 | -241 | -357 |
| 17 | Female | 54 | Sigmoid colon | T1N0M0 | G1 | No | - | - | - | - |
| 18 | Female | 72 | Cecum | T2N0M1a | G4 | No | - | - | - | - |
| 19 | Female | 76 | Cecum | T3N1BM0 | G3 | Yes | Mesenteric vein | 41 | 115 | 74 |
| 20 | Female | 64 | Sigmoid colon | T2N0M0 | G1 | No | - | - | - | - |

Abbreviations: VTE = venous thromboembolism, TNM staging = tumor classification used according Americal Joint Committee on Cancer (AJCC) with T: size or direct extent of the primary tumor, N: spread to regional lymph nodes, and M: presence of distant metastasis. Grade = histological grade with G1: welll differentiated, G2: moderately differentiated, G3: poorly differentiated, G4 undifferentiated [26].
